# Cohort profile: the Dynamics of Family Conflict (FamC) study in Norway

**DOI:** 10.1136/bmjopen-2023-080772

**Published:** 2024-08-22

**Authors:** Linda Larsen, Nelli Buchmann, Maria Morbech, Tonje Holt, Espen Roysamb, Maren Sand Helland

**Affiliations:** 1 Norwegian Institute of Public Health, Oslo, Norway; 2 Promenta Research Center, Department of Psychology, University of Oslo, Oslo, Norway

**Keywords:** MENTAL HEALTH, Child & adolescent psychiatry, PUBLIC HEALTH

## Abstract

**Abstract:**

**Purpose:**

The Dynamics of Family Conflict (FamC) prospective cohort study was set up to investigate how and why interparental conflicts and family relations develop over time, and in which contexts which types of conflicts and relations are most negative for which children. FamC focuses on the family within a scope spanning macrolevel as well as microlevel processes.

**Participants:**

Families were recruited from MoBa (pilot project) and family counselling offices across Norway when parents attended parental counselling, therapy or mandatory mediation in relation to parental relationship dissolution. All families were thus experiencing challenges and/or going through a family transition. Families were eligible for the study if parents had at least one joint child between 0 and 16 years. Both parents and up to five children from the same family could participate. A total of 2871 families were recruited (participation rate wave 1: 78%) and an estimated 55% of parents (based on wave 1 data) were divorced/separated. Additional data were obtained from therapists/mediators at the family counselling offices about the family, and childcare or schoolteachers provided data on the youngest (0–6 years) children.

**Findings to date:**

Results show that interparental conflict patterns vary with family constellation. Interparental conflict severity is inversely related to the discrepancy between child-reported and parent-reported child reactions to interparental conflicts, and child-self-reported reactions are higher relative to parent-reported child reactions. Other findings show that family characteristics (eg, the number and age of children in the family and financial difficulties) are predictive of the type of residence arrangement that parents practice.

**Future plans:**

The cohort is ideally suited for cross-cultural comparisons and further examination of family processes and dynamics including parent repartnering, step-parents and new family members, associations between different family constellations and child adjustment, and fathering, father–child relationship and child adjustment. There are plans for further follow-up data collection.

**STRENGTHS AND LIMITATIONS OF THIS STUDY**

- Dynamics of Family Conflict (FamC) is unique in that it employs a longitudinal and multireporter design where mothers, fathers and up to five children from the same family can participate, allowing for a better understanding of the dynamic nature of the family system and how this relates to child adjustment over time.
- The FamC cohort is large and considered well-suited to studying interparent conflict and family dynamics.
- The inclusion of children down to 7 years is an important feature rarely seen in family research.
- The FamC surveys are extensive, cover many domains and include nuanced measures of interparental conflict and living arrangements for children whose parents are separated.
- Due to families being recruited from family counselling offices, the study population is not representative of the general population in Norway.

## Introduction

 The link between interparental conflict and child adjustment and development is long-recognised in the literature with findings showing that conflicts that are long-lasting, intense, hostile and child related are particularly detrimental for children.^1^,^3^ However, knowledge is still lacking about (1) how and why interparental conflicts develop over time and (2) in which contexts which types of conflicts are detrimental for which children. Thus, the overarching aim of the Dynamics of Family Conflict (FamC) study was to develop a cohort that enables investigations of family dynamics and effects on family members, particularly the children, within a scope spanning macro (eg, Norwegian welfare context, family policy, living and working conditions) as well as micro (eg, family structure, residence arrangements, parent and child characteristics) level processes. In this cohort profile paper, we describe the background for the FamC study, give an overview of the cohort and detail the study design to provide a basis for future analyses using the cohort. We also present a brief overview of some of the findings from the cohort to date.

Using the emotional security theory as a theoretical backdrop,^4^ Cummings *et al* have demonstrated the importance of distinguishing between different forms of interparental conflict, such as destructive and constructive conflict, and the differential effects of these on child development and mental well-being.^5 6^ At a more nuanced level, the core elements of the complex and multidimensional construct of interparental conflict include frequency, intensity, duration, pattern, mode of expression and degree and mode of resolution.^7 8^ In recent years, the importance of conflict resolution for understanding the effects of interparental conflict on the family has been highlighted, with research showing that more constructive conflict resolution, like compromise or agreeing to disagree, may buffer against the negative effects of interparental conflict on child adjustment.^9 10^ Theoretically, FamC leans on family systems theory^11 12^ and Bronfenbrenner’s ecological model of child development^13^ as well as emotional security theory.^4^ The family is thus viewed as a dynamic internal system, where different subsystems, for example, the parent–child subsystem and the sibling subsystem, are mutually affected by each other and as a system that is embedded in a larger, societal context. Being carried out in a Nordic welfare society, FamC is well situated to investigate how family relations are related to the welfare reforms and systems in which they are embedded.

In studying family dynamics, FamC has the advantage of using a multireporter design, including the perspectives of mothers, fathers and children above 7 years within each participating family. These perspectives are supplemented by reports from the family therapists that recruited the families, and childcare personnel or schoolteachers’ reports about the well-being of children below 7 years. In addition, linkages are made to health and demographic data on all recruited families from national registries. Pertaining to interparental conflicts particularly, a multireporter perspective in family research is important given the dynamic nature of the family system.^11^ Furthermore, a major focus of FamC is to highlight the impact of family dynamics on children. As children have unique perspectives on family life, interpretations of family relations and their own agencies,^14^ including the children’s own perspectives is an important attribute of the current study cohort. Furthermore, linking the FamC data to national registries is another important attribute, as information about sociodemographic characteristics and health is gathered without the parents having to provide this information. This may help to prevent attrition as the questionnaires are shortened.

Another backdrop of FamC is the changing family patterns seen in Norway, as in most western countries, where a large number of children grow up in two homes and with parents living apart.^15^ With an increasing number of children spending equal or close to equal time in both parents’ homes, it is important to understand how children are affected by interparental conflicts and other aspects of family dynamics across different living or residence arrangements. Norway is a well-suited country for studying family dynamics across residence arrangements, as mandatory mediation is provided to all families when parents are about to move apart. By recruiting families from the mandatory mediation, FamC is in a position to control for important confounders hypothesised to impact the mental well-being of children in postseparation families.

Taking a cross-cultural perspective, family dynamics may be different in the Nordic social democratic welfare context than in a more liberal welfare context such as North America. However, there is also evidence of some similarities. For example, in both Norway and the USA, there are negative associations between single motherhood and child adjustment with comparable moderation effects of family economic resources.^16^ Yet, little is known about whether findings regarding interparental conflict from North American studies generalise to Norway. Thus, to better understand the complex relations between interparental conflict and child outcomes under the current societal context for family life, the main scientific aim of FamC was to generate more knowledge about the interplay between macrolevel and microlevel family processes enhancing the well-being of different families and children across different contextual settings. To achieve this aim, a large sample of families from across Norway was recruited and survey data from both parents and children on a range of variables including family dynamics, interparental conflict and parent and child mental health and well-being were collected. Information about parents’ cohabitation status and residence arrangements for their children (only parents living apart) was also obtained, as were linkages to demographic and health data from national registries. Collectively, these data allow for the following specific project aims to be addressed:

1. Increase our understanding of how parental conflict relates to child adjustment in the context of a Nordic welfare society.
2. Identify mechanisms involved in the association between family structure and child development.
3. Identify the most harmful patterns of interparental conflict for children.
4. Increase our understanding of family mediators and moderators of the association between parental conflict and child adjustment.

These aims have been expanded on to include new and related projects addressing the following central aims: (1) Identifying conditions to secure the well-being of children across different residence arrangements when parents live apart; (2) Increasing our knowledge about children’s agency when parents live apart; (3) Increasing our knowledge about family relations and gender equality, (4) Providing knowledge about possible consequences of the COVID-19 measures for both families in general and children in vulnerable families in particular and (5) Identifying and securing proactive services for vulnerable families by using the experiences from the COVID-19 pandemic. Although the COVID-19 pandemic was not initially a focus of FamC, the cohort is ideally suited to addressing questions pertaining to the heightened concern for vulnerable families during and after the pandemic as it comprises help-seeking families and families going through divorce/separation and includes prepandemic, peripandemic and postpandemic measures. In the future, there are plans for even further follow-up data collection and expanding the focus to fathering, father–child relationship, new postseparation families and child adjustment along with cross-cultural comparison studies exploring family dynamics.

## Cohort description

### Location

FamC is a longitudinal survey study situated in Norway. Norway is geographically diverse and nearly half of the country’s inhabitants live in the far south, around the capital Oslo or near the coastline in other large cities. Across the Nordic countries, equality and social cohesion are fundamental values of the respective governments, and policy is aimed at reducing economic and social differences. There is a strong emphasis on labour participation and strengthening welfare services such as healthcare and education. The Nordic countries rank among the highest in the world on gender equality.^17^ The equalisation of care and parenting responsibility, combined with increased female participation in the paid labour force, has been an explicit policy goal across the Nordic countries. For example, Norway has low-cost childcare for all children combined with a generous parental leave scheme for mothers and fathers. Since its introduction in 1993, the quota for paid paternity leave has increased from 4 weeks to 10–15 weeks, with over 70% of fathers using their full paternity leave.^18^ Moreover, education (including university) is free of charge in Norway (with only a few exceptions). Children start school the year they turn 6 and most attend secular public schools. Children generally attend the school closest to where they live, at least up to upper secondary school (ages 16–19), and while the school day is relatively short (about 8:30–14:00 hours), schools provide after-school care up to grade 4. The social welfare system in Norway is generally more comprehensive and has more egalitarian cultural attitudes compared with, for example, North America or the UK.^19^ These differing cultural contexts may lead to variations in how interparental conflict varies, is dealt with and impacts family members across these societies.

The Norwegian Directorate for Children, Youth and Family Affairs (Bufdir) is responsible for state-funded child welfare services and family counselling provided by therapists and psychologists in family counselling offices across the country. This is considered a low-threshold service free of charge to families experiencing difficulties in the parental relationship and/or the parenting role. The service also facilitates mandatory mediation when parents with children under the age of 16 years divorce/separate. Children also have the right to have their opinions heard when their parents are separating, and they have the opportunity to speak to a mediator if the parents allow it. At the time of writing, Norway is the only country in the world where mediation is mandatory.

### Recruitment and data collection

Recruitment took place when parents attended parental counselling, therapy or mediation at 1 of 37 different family counselling offices across Norway (except the most northern parts) and spanned from December 2017 to August 2019. Therapists from these offices were trained to inform and invite all families to the study, irrespective of whether the parents lived together or not. Families were eligibility for the study if parents had at least one joint child aged 0–16 years, and both parents and up to five children from the family could be part of the study. We note that a small number of single/widowed parents were also included.

Families were invited to participate in wave 1 (W1) shortly after recruitment. Parents and children 12 years and older participated in each wave (six in total) by answering online questionnaires. For children aged 7–11 years, a revised form of the youth questionnaire was administered as a structured interview by trained interviewers. At W1 and W2 (until the onset of the COVID-19 pandemic), these interviews were conducted in the child’s home or in another suitable location close to the home. However, after this point, the interviews for the remainder of W2, W3 and W6 were conducted digitally via video link. There were no interviews at W4 and W5. Childcare personnel or schoolteachers (if the child had already started school; henceforth teacher) answered a short questionnaire about children aged 0–6 years at W1, W2 and W5 only while therapists/mediators at the family counselling offices answered a questionnaire about the family at W1 only. A total of 1947 mothers, 1477 fathers, 1414 children and 783 teachers participated in one or more waves across all data collection waves. See table 1 for an overview of family-level and individual-level participation and online supplemental table 1 for further details about participation rates.

**Table 1:** Overview of family, family member, teacher and therapist participation at each data collection wave

	W1	W2	W3	W4	W5	W6
	n	n	n	n	n	n
Family level						
Families in total (any family member or teacher)	2313	1215	987	836	972	934
Families in total (any family member)	2231	1086	987	836	817	934
Families in total (teacher report only)	82	129	–	–	155	–
Both parents (no children)	388	78	51	36	34	44
Both parents+at least one child	588	125	77	72	53	40
One parent only (no children)	959	538	520	515	427	342
One parent only+at least one child	234	190	157	99	147	210
One or more children (no parents)	62	155	182	114	156	298
Participant level						
Mothers*	1792	726	613	552	508	479
Fathers	1353	408	319	278	240	241
Children†	919	537	482	296‡	421§	694
Teachers¶	647	297	–	–	270	–
Therapists	1996	–	–	–	–	–

Note. Participation refers to answering questionnaires. Family -level participation can be operationalised in different ways. For instance, at W1, there were 2313 families that participated based on (1) at least one family member answering the questionnaire (n=2231: Families in total (any family member)) or (2) a teacher answering the questionnaire about a child aged 0–6 years in the family (n=82: Families in total (teacher report only)).

* Mothers are inclusive of both parents from the same-sex mother families.

† Children 7 years or older.

‡ Children 12 years or older.

§ Children 11 years or older.

¶ Teacher report on children aged 0–6 years.

**Figure 1:**
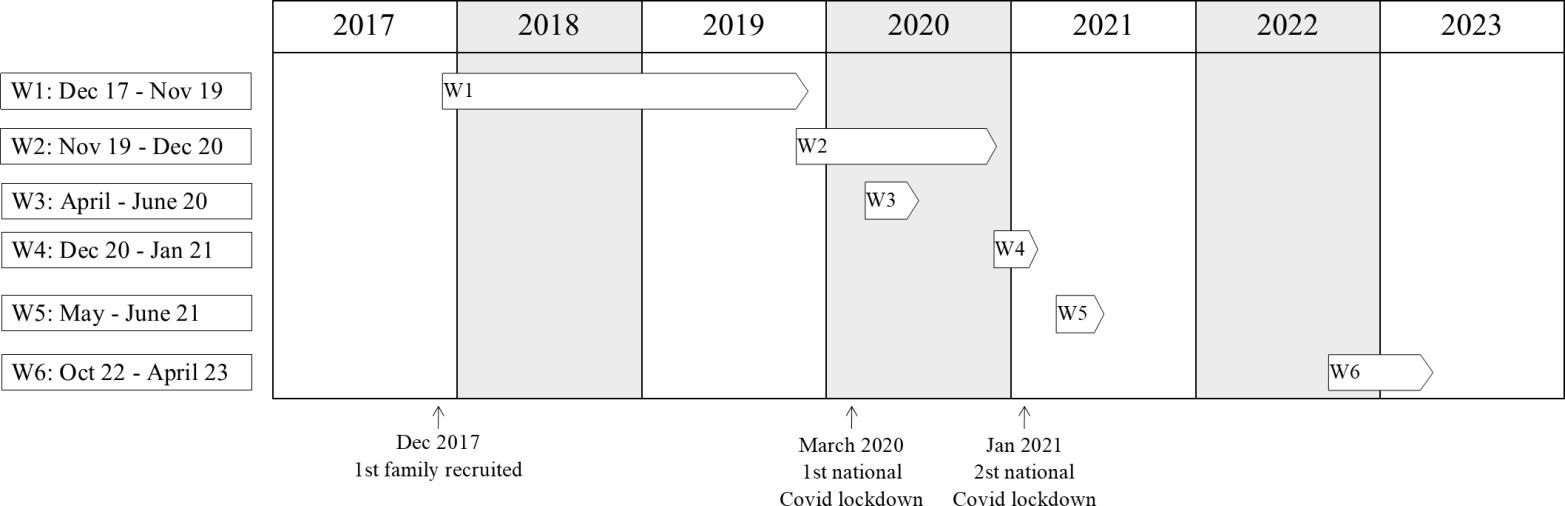
Overview of data collection waves.

At the time of writing, six data collection waves have been completed as depicted in figure 1. Families were invited to W2 approximately 18–24 months after they were invited to W1 (irrespective if they had answered the W1 survey). In March 2020, 5 months after W2 was initiated, the unprecedented COVID-19 pandemic caused Norway to go into lockdown and W2 was momentarily suspended. It was decided to initiate W3 to explore the effects of the pandemic on the families in the study. In April 2020, all families were invited to W3 irrespective of whether they had already been invited to W2. Steps were taken to keep the duration between W1 and W2 as similar as possible across participants, and thus, some participants were invited to W2 and W3 at the same time while others were invited to W3 before W2. With the enduring COVID-19 pandemic, Norway went into a second lockdown at the end of 2020, and W4 was initiated to follow the consequences for families more long-term. Finally, families were invited to W5 and subsequently W6. W1 (December 2017–January 2020), W2 (November 2019–December 2020), W5 (May–July 2021) and W6 (October 2022–April 2023) are considered part of the main FamC while W3 (April–June 2020) and W4 (December 2020–January 2021) are considered additional COVID-19 waves.

### Participants and participation across data collection waves

A total of 2871 families (including 20 same-sex mother families) were recruited, but regrettably, it is not known how many families declined to be included in the study when informed about it. Of the recruited families, 1031 families (36%) were recruited when they attended mandatory mediation due to parental relationship dissolution, 1184 (41%) when they attended family therapy, and 656 (23%) when they attended counselling to improve coparent cooperation after separation.

A total of 3145 parents from 2169 families participated (ie, contributed with data by answering the questionnaire) at W1, and more mothers than fathers participated. Mothers were on average, just over 3 years younger than fathers. A larger proportion of fathers were in part-time or full-time employment than mothers, but mothers and fathers were similar in their report about how the family managed financially. See table 2 for further details. Further, a total of 919 children from 684 families participated in W1. These children were on average 10.62 years (SD=2.55) and slightly more girls (52.45%) than boys participated. More than 95% of children were born in Norway and most (68.12%) had parents who were divorced/separated or about to separate. Moreover, 647 teachers provided data on the youngest children (0–6 years), who were on average 4 years (SD=1.74) and almost half (46.37%) were girls. Most teachers were employed in a childcare setting (87.79%).

**Table 2:** Sample characteristics for parents at wave 1 based on parent self-report

	Mothers (n=1792)	Fathers (n=1353)
	n (%)	n (%)
Age in years (M, SD)	36.95 (6.91)	40.20 (7.50)
Employment		
Full-time employment (≥80%)	1024 (57.24)	1106 (82.41)
Part-time employment (<80%)	228 (12.74)	61 (4.55)
Other (studying, parental leave, sick leave, job seeking, other)	537 (30.02)	175 (13.04)
Birth country		
Norway	1614 (90.07)	1225 (90.54)
Other	178 (9.93)	128 (9.46)
Family economy		
We manage well/very well	1124 (62.90)	850 (63.43)
We manage	578 (32.34)	416 (31.04)
We manage poorly/very poorly	85 (4.76)	74 (5.52)
Case type		
Mediation	567 (32.14)	457 (33.78)
Family therapy	811 (45.26)	603 (44.57)
Parent cooperation	405 (22.60)	293 (21.66)
Cohabitation situation		
Living together	686 (38.58)	538 (40.18)
Separating/living apart <6 months	422 (23.73)	298 (22.26)
Living apart >6 months	621 (34.93)	476 (35.55)
Never lived together, widow, sole custody	49 (2.76)	27 (2.02)
Number of children with the other parent (M, SD)	1.76 (0.74)	1.79 (0.75)
Number of children in the family participating in the study		
1	886 (49.64)	636 (47.25)
2	760 (42.58)	596 (44.28)
3	129 (7.23)	104 (7.73)
≥4	10 (0.56)	10 (0.74)
Relationship duration (only parents living together) (M, SD)	11.32 (6.36)	11.66 (6.28)
Duration since separation (only parents living apart) (M, SD)	3.88 (3.17)	3.11 (3.11)

Note. Mothers are inclusive of both parents from the same-sex mother families.

**Figure 2:**
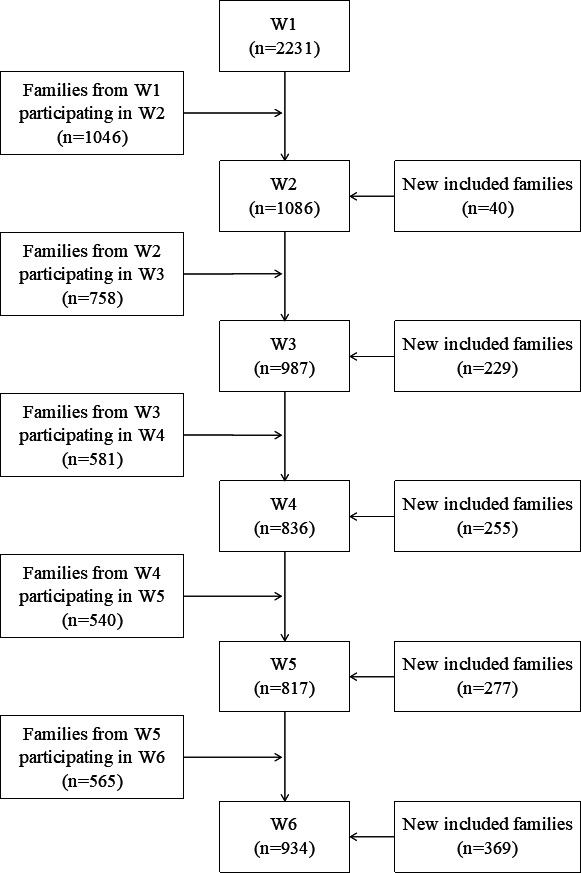
Family participation in each data collection wave. Families participating in W1 and all new included families at W2–W6 add to 3401 families. This exceeds the number of families recruited to the study (n=2871) because some families are counted more than once when adding up ‘new included families’. New included families in each wave (eg, **W4**) are those families that did not participate in the previous wave (ie, **W3**). These families may not have participated up until this point, or they may have participated in earlier waves but not in the one immediately preceding the current wave. By family participation, we mean that any family member answered the questionnaire.

We observed attrition across the subsequent data collection waves. This was most pronounced from W1 to W2, and particularly for fathers, who moreover showed the highest attrition rate across the entire study duration. Attrition from W1 to W2 was related to the parents’ living situation (ie, live together or live apart) such that among the parents who did not live together, the attrition rate was higher (ie, they only participated in wave 1). Moreover, for mothers only, attrition was related to how many children she had with the father in that the attrition rate was higher among families with only one child. The parents’ work situation, country of birth, life satisfaction and level of interparental conflict were not predictive of attrition from W1 to W2. In terms of family participation, the participation rate was reduced to around half at W2 and W3 compared with W1 for families in total (based on any family member participating) and then remained relatively stable throughout. The highest family attrition rate was for families where both parents participated with or without any of their children while the lowest attrition rate was for families where only one parent and at least one child participated (see table 1). Figure 2 presents a flow diagram of family participation, which also shows how many families from each data collection wave participated in the subsequent wave and how many new families (ie, families that had not participated in the previous wave) participated. For instance, of the 2871 recruited families, there were 2231 families where the mother and/or father answered the questionnaire with or without any of their children at W1. Of these W1 participating families, 1046 families also participated in W2 along with 40 new families that were originally recruited but had not participated in W1. Thus, a total of 1086 families participated in W2.

**Figure 3:**
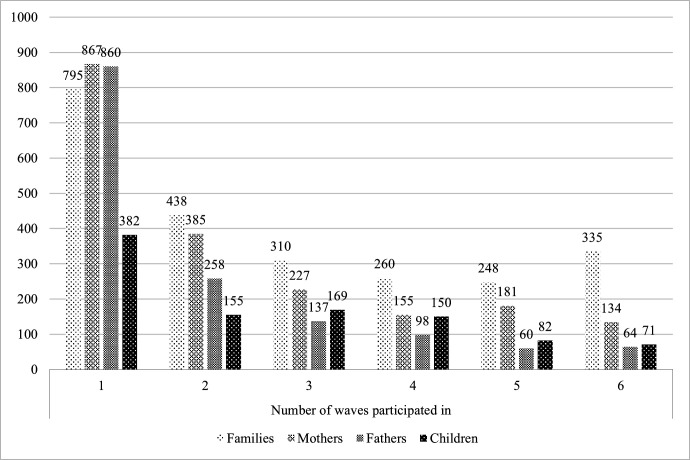
Number of waves participated in by families, mothers, fathers and children.

Of the 2871 families recruited, there were 2386 families that participated (ie, contributed with data by answering the questionnaire) at one or more waves and around one-third participated in only one wave (n=795, 33.32%). The number of families participating in an increasing number of waves gradually declined and specifically, 438 (18.36%) families participated in 2 out of 6 waves, 310 (12.99%) families participated in 3 out of 6 waves, 260 (10.90%) families participated in 4 out of 6 waves, 248 (10.39%) families participated in 5 out of 6 waves and 335 (14.04%) families participated in all 6 waves. Figure 3 presents an overview of the number of waves that families (ie, any family member), mothers, fathers and children (ie, at least one child in the family participating) participated in.

In a large proportion of the sampled families, parents were divorced/separated or about to separate. Both parents were asked about their cohabitation status and at W1, 971 parent dyads (out of 2161 parent dyads where one or both responded to the question about cohabitation status) reported that they were living together (including parents still living together but about to move apart) and 1190 were living apart, at W2, 344 (out of 905) were living together and 561 were living apart, at W5, 239 were living together (out of 635) and 396 were living apart, and finally, at W6, 226 (out of 613) were living together and 387 were living apart. Given the longitudinal study design, it was possible to estimate how many parent dyads were initially living together but separated at a later point during the study. Out of 984 families where parents were living together or about to move apart at W1, there were 174 that divorced/separated at a later point and most (n=138) divorced/separated between W1 and W2 while the remaining divorced/separated by W5.

### Study domains and pretesting

The surveys in FamC are extensive and cover many domains relevant to addressing the study aims and a detailed overview of the assessment domains and variables for parents, children and teachers, respectively, is available in online supplemental tables 2–4. Parents and children were asked about many of the same domains including parental conflict, experience with mediation (if applicable), parent and child mental health and well-being, family life and the parent–child relationship. Key domains, such as interparental conflict, and parent and child mental health and well-being, were included in all data collection waves to allow for investigations of development over time while certain demographic and background variables were only included at W1 and/or W2. Domains were assessed with already existing and validated questionnaire scales or selected items from other Norwegian studies such as the Mother, Father and Child Cohort Study (MoBa)^20^ or the Tracking Opportunities and Problems Study.^21^ When validated questionnaires were not available, the research group developed appropriate items (estimated to be approximately 10% but varied slightly across data collection waves). For the COVID-19 waves, a substantially higher proportion of researcher-developed items were used both to assess context-specific pandemic effects and also because already validated questionnaires were not available. Researcher-developed items were included to assess, for example, experience with mediation and satisfaction with residence arrangement and several domains specific to the experience of the COVID-19 pandemic.

Part of the study preparation phase involved pretesting the questionnaires on parents and children. For the children, particular effort was put into the pretesting as children as young as 7 years could participate. A whole day with pretesting of the structured interview was arranged for a group of children 7–11 years. The children gave feedback on questions they did not understand, the length and the illustration tools used during the interviews. For the older children, the digital version of the questionnaire was tested in a school class in Oslo (12-year-old children). The pretesting led to certain questions being revised, and short explanations were added for questions that were hard to understand or confusing for the children.

Subsequently, the parent and child questionnaires in FamC were tested, and quality assured in a pilot study using a sample of 398 families. The focus was particularly on establishing the reliability and validity of the measures used to assess the key domains. The majority (n=381) of the pilot sample was recruited from MoBa,^20^ a longitudinal, prospective population-based cohort study that until now has followed children from prebirth until adolescence. Over 90 000 pregnant women were recruited from all over Norway from 1998 to 2008, and more than 70 000 fathers participated. Altogether, a random subsample of 2500 families from MoBa was invited to participate in the FamC pilot in 2015 and 2016 if (a) the parents were living together and (b) they had a child together who turned 11 years during the year of recruitment. Invitations and consent forms for the FamC pilot were sent by mail to all participants. Both parents had to provide written consent for their child to participate. Each family member’s questionnaire was sent to the family after the consent form was returned. The children were provided information about the study in a separate letter. Since the sample recruited from MoBa is rather homogeneous and well-functioning, a total of 17 families with a child aged 11–16 years and undergoing mediation at a family counselling office were added.

### Registry linkages

FamC already has existing consent to link to registry data on all recruited families. This adds a valuable dimension to the FamC data foundation with the possibility of following the families and its members long-term. Specifically, FamC links to registry data from Statistics Norway (SSB), the Norwegian Control and Payment of Health Reimbursements Database (KUHR), the Norwegian Patient Registry and the Norwegian Labour and Welfare Administration (NAV). Variables accessed from these registries include but are not limited to, employment, profession, parental income, household income, child welfare services (eg, type of intervention and duration), demographics (eg, country of origin), social security benefits (eg, unemployment or sickness benefits, benefits for caring for a sick child, disability pension), and mental health and other diagnosis (eg, anxiety and attention deficit hyperactivity disorder).

### Patient and public involvement

To enhance the feasibility and impact of FamC, a reference group was put together comprising high-expertise researchers within the fields of family research and developmental psychology and clinicians from the family counselling offices. The reference group was involved in discussions regarding the study design, the development of research questions and provided theoretical, methodological and practical guidance on the project. The perspective of participants is deemed important to improve the quality, validity and ethical conduct of FamC. Therefore, the participants can provide feedback on the questionnaires (or give other relevant information) using an open-text field at the end of the survey. The implementation of study results in the practice field is undertaken in collaboration with Bufdir, and family counselling offices across Norway. Popular science summaries are provided on the project homepage for participants and the general population, including summaries specifically for the children.

### Findings to date

More than 10 articles based on data from FamC have been published in national and international peer-reviewed journals. A complete list of all publications is available at this website: https://www.fhi.no/op/studier/familieforsk/resultater-fra-familieforsk/.

### Scale development and validation

Three articles have examined the psychometric properties of some of the primary scales and measures used in the study. Helland *et al*^22^ developed and validated short forms of the conflict strategy scales from the Conflicts and Problems-Solving Scales (CPS) developed by Kerig,^23^ a widely used parent-report measure assessing major dimensions of interparental conflict (eg, conflict strategies, conflict content and conflict resolution) while Larsen *et al*^24^ validated the conflict resolution scale from the CPS and developed and validated a short form of the same scale. Holt *et al*^25^ developed and validated short forms of the properties subscales (ie, frequency, intensity, resolution, child content and triangulation) from The Children’s Perception of the Interparental Conflict Scale^26^ and a modified version of the reactions subscales from The Security in the Interparental Subsystem.^27^ These measures assess children’s reactions to interparental conflict and use child self-report.

### Interparental conflict, family constellations and residence arrangements

Results from some of the other empirical articles show that patterns of interparental conflict vary with family constellation; parents living together have more frequent conflicts but are better at resolving these compared with parents living apart. For parents living apart though, those in more complex family constellations (ie, where one or both parents have repartnered) have more destructive conflict behaviours and poorer conflict resolution compared with less complex family constellations (ie, neither parent has repartnered).^28^ Focusing specifically on families where parents live apart, Morbech *et al*^29^ explored parent characteristics in relation to what type of residence arrangement parents chose for their child, with results showing an increased likelihood of practising a 50/50 arrangement (ie, equal time with both parents) when parents had more and older children, had fewer financial difficulties and had separated more recently. This study is unique in that it applied a nuanced set of four residence arrangements, unlike previous research that typically focuses on shared residence vs sole residence. Two qualitative studies by Eikrem and Andenœs^30^ and Eikrem and Jevne^31^ have also been published. Eikrem and Jevne conducted individual, in-depth, semistructured interviews with a small number of separating/separated parents and their children with the aim of exploring parenting in transition as part of a continuous parental relationship, and parents and children’s experiences with mandatory mediation. A main finding was that co-parenting after separation requires hard work even in no-conflict or low-conflict separations, and parents are motivated to work hard for the sake of the children and by maintaining a close relationship between each parent and the child independent of the marital relationship.^31^

### Children’s agency

Two other articles have focused on children’s perspectives and agency. Sunde *et al*^32^ identified emerging patterns in children’s opinions about different aspects of their living situation when their parents were about to or had already separated. Stability (eg, staying at the same school, meeting friends when at both parents’ homes) and openness (eg, being able to talk about mum (dad) when at dad’s (mum’s) house) were the most important aspects for the children while the practical terms of their living situation (eg, same house rules apply at both parents’ homes) were less important for children. Moreover, children’s age appeared more important than interparental conflict in predicting which aspects children had clear opinions about. Tveit *et al*^33^ explored how central family processes of conflict and attachment affected the extent to which children were invited to express their views, both in mediation and the home when their parents are separated/separating. There was a significant association between having been invited to participate in mediation and having been asked about their views by their parents. Moreover, attachment security to mothers predicted child invitation to participate in mediation while older children were more likely to have been invited to share their views in mediation and at home.

### COVID-19 pandemic

Principal COVID-19-related findings include a documented effect of increased parenting stress, but parents did not show an increase in mental health symptoms or in destructive conflict strategies due to the first phase of the pandemic.^34^ Children on the other hand reported fewer emotional reactions in the first phase of the pandemic (eg, feeling sad, unsafe and lonely), but more somatic reactions (eg, headaches and trouble sleeping) compared with before the pandemic, and their experience of stress and instability in the family was a significant predictor of their emotional, somatic/cognitive and worry reactions to the pandemic.^35^ Regarding the families’ need for welfare services during the first phase of the pandemic, around half of the sample reported such a need. Parental psychological problems and destructive conflicts were related to whether the families needed support services during this period. Further, there was a lower probability that families with more conflicts and less family support contacted relevant services despite needing support.^36^

Moreover, seven master’s theses have used data from FamC, and three PhDs are currently underway. Other results pertaining to related projects using FamC data and funded by Bufdir have been delivered in reports in Norwegian.

### Strengths and limitations

The FamC prospective cohort study has three principal strengths. First, FamC is set in a Nordic welfare context and is concerned with families living in this context. Thus, it differs from most former research on family dynamics and interparental conflict, and effects on child adjustment that has been conducted in more market-oriented welfare contexts such as North America. In the Nordic countries, there is a clear focus on gender equality between parents for childcare as well as economic responsibility. FamC’s contextual placement enables a new and important perspective to family research and is unique in focusing on macrolevel and microlevel aspects and perspectives of interparental conflict.

Second, FamC employs an ambitious research design. It is a multireporter study with data collection from both parents and several children from the same family. Moreover, children as young as 7 years are eligible to participate. This is rare in family research. Substantial efforts were put into developing the survey for the younger children (who were interviewed), and it is an important component of the study as well as a contribution to the research field by placing such emphasis on children’s own report (or voice) on family matters that concern them and their own mental health and well-being. The data foundation was further strengthened by including data from family counselling therapists/mediators and teachers, and linkages to contextual and background data from national registries and not least, by tracking the families over time. Additionally, FamC sought to sample families that are heterogeneous in terms of their constellation and the level of difficulties experienced, and they come from both urban and rural areas of Norway.

Third, the surveys used in FamC cover many different domains and are extensive in nature allowing for nuanced investigations of the complex interplay between factors to understanding family dynamics, interparental conflict and relations with child adjustment and parent mental health and well-being. A particularly noteworthy feature of FamC is that it covers different conflict strategies and patterns, the type and level of conflict resolution, conflict topics or content, in addition to the frequency/chronicity of interparental conflicts. Detailed information is also provided by parents living apart about the residence arrangement they practice, and children and parents are asked about their experiences with the chosen arrangement. FamC thus provides a very real representation of the dynamics of residence arrangements in two-thirds of the sample where parents live apart. This contrasts with previous longitudinal studies that tend to assume a consistency in the chosen residence arrangements over time.

FamC also has some important limitations that deserve mentioning. First, as with any longitudinal study, we observed attrition during the study period. The study may have been particularly susceptible to this given the sampled families experienced some level of difficulty in the parental relationship or with family functioning. Yet, the sample size is still relatively unmatched for studies focusing on interparental conflict. Second, the FamC cohort is not representative of the general population in Norway, something that affects the generalisability of the study results. This is because the families were recruited when they sought help or attended mandatory mediation at a family counselling office. The cohort can, therefore, be characterised as help-seeking and having some challenges with respect to family dynamics and relations, affecting its representativeness of the general Norwegian population. Third, although efforts were made to recruit families with different cultural backgrounds, this unfortunately proved futile in boosting the cultural diversity of the sample. Finally, at the initial data collection (W1) sparse information was collected about new partners from those parents who had repartnered. This is an important dimension to cover and was addressed in subsequent data collection waves.

## supplementary material

10.1136/bmjopen-2023-080772 online supplemental file 1

## Data Availability

Data are available on reasonable request.
