# Supplementary material for: Cohort profile: the Dynamics of Family Conflict (FamC) study in Norway
Source: BMJ Open. 2024 Aug 22;14(8):e080772. doi: 10.1136/bmjopen-2023-080772 (PMC11344520; doi:10.1136/bmjopen-2023-080772)
Supplement: online supplemental file 1 [file bmjopen-14-8-s001.pdf]

Article title:

COHORT PROFILE: THE DYNAMICS OF FAMILY CONFLICT (FAMC) STUDY IN NORWAY

Author information:

Linda Larsen[1] https://orcid.org/0000-0002-6910-4946

Nelli Buchmann[1]

Maria Morbech[1] https://orcid.org/0000-0001-8451-4436

Tonje Holt[1] https://orcid.org/0000-0002-9057-4010

Espen Røysamb[1 2] https://orcid.org/0000-0001-5133-7170

Maren Sand Helland[1] https://orcid.org/0000-0001-9728-4094

[1] Division of Mental & Physical Health, Norwegian Institute of Public Health, Oslo, Norway

[2] Promenta Research Center, Department of Psychology, University of Oslo, Oslo, Norway

Corresponding author:

Linda Larsen

Mobile: +47 93964849

Email: linda.larsen@fhi.no

Supplemental Table 1

Overview of Participants and Participation Rate

	Total	W1	W2	W3	W4	W5	W6
Participants	*n (%)*	*n (%)*	*n (%)*	*n (%)*	*n (%)*	*n (%)*	*n (%)*
Mothers	1947 (100)	1792 (92)	726 (37)	613 (31)	552 (28)	508 (26)	479 (25)
Fathers	1477 (100)	1353 (92)	408 (28)	319 (22)	278 (19)	240 (16)	241 (16)
Children [a]	1414 (100)	919 (65)	537 (38)	482 (34)	296 (21) [c]	421 (30) [d]	694 (49)
Teachers [b]	783 (100)	647 (83)	297 (38)	-	-	270 (34)	-

Note. Participation rate is based on the total number of participants at one or more waves across all data collection waves. For example, 1947 mothers participated at one or more waves across the six data collection waves and based on this, the participation rate for mothers at W1 was 92%. Child and teacher participation and participation rates should be interpreted with caution as some children have changed category, that is, they have moved from teacher report to participating through self-report (i.e., structured interview) as they have turned 7 years.

[a] Children 7 years or older.

[b] Teacher report on children aged 0-6 years.

[c] Children 12 years or older.

[d] Children 11 years or older.

Supplemental Table 2

Overview of the Study Domains and Instruments in the Parent Surveys

Domain	Instruments and description	W1	W2	W3	W4	W5	W6
Interparental conflict	CPS – Conflict strategies, content, frequency, resolution, and relationship satisfaction	mf	mf	mf	mf	mf	mf
	Current experiences of problems in the co-parenting relationship	mf	mf	-	-	mf	mf
	Disagreements about custody and parental responsibilities (from a mediation screening tool used by the Family Welfare Service) [a]	mf	-	-	-	-	mf
	Frequency and mode of contact with the other parent [a]	-	mf	-	-	mf	mf
	Strains and concerns in relation to the other parent (e.g., drugs, alcohol, violence) (from a mediation screening tool used by the Family Welfare Service)	mf	-	-	-	-	-
	Support from the other parent (TOPP)	mf	mf [b]	mf	mf	mf [b]	mf [b]
	Trust between parents and experience of custody disputes at court (items from a mediation screening tool used by the Family Welfare Service) [b]	mf	mf	-	-	mf	mf
Mediation [b]	Experience with mediation	mf	mf	-	-	-	-
	Type of time-sharing arrangement [b]	mf	mf	-	-	mf	mf
	Experiences with time-sharing arrangement and extent to which parents follow agreement [b]	-	mf	-	-	mf	mf
Child development and mental health [a]	SDQ – Psychological symptoms of mental health	m	m	mf	mf	mf	mf
	ASQ – Child development	m	m	-	-	m	-
	SSIS – Social skill	-	f	-	-	f	mf
	SIS – Child coping strategies during interparental conflict	f	f	-	-	mf	mf
	EAS – Emotionality or distress, degree of activity, sociability, and shyness	f	f	-	-	f	-
	Illness/disability (psychological, physical and/or cognitive)	mf	mf	-	-	mf	mf
	Sleep and appetite	-	mf	mf	mf	mf	mf
	School results maths and Norwegian (FORM)	m	m	-	-	m	mf
	School adjustment and school absence	-	-	-	-	-	mf
	Special education needs/arrangements (MoBa)	m	m	-	-	m	mf
Parent-child relationship [a]	CPRS – Parent perception of relationship with the child	mf	mf	-	-	mf	mf
	PEQ – Parent-child conflict	mf	mf	-	-	mf	mf
Family life	FAD – Family functioning	-	mf	-	-	mf	mf
Parent mental health and wellbeing	SWLS – Satisfaction with life	mf	mf	mf	mf	mf	mf
	SCL-8 – Mental distress (symptoms of anxiety and depression)	mf	mf	mf	mf	mf	mf
	GSAQ – Sleep quality	-	mf	mf	mf	mf	mf
	AUDIT – Risky and hazardous alcohol/drug use	mf	mf	mf	mf	mf	mf
	FSSQ – Functional social support	mf	mf	mf	mf	mf	mf
	Sources for prolonged stress and strain (TOPP)	-	mf	-	-	mf	-

	BFI-44 – Personality	mf	-	-	-	-	-
	Parent’s own upbringing and family environment (MoBa) [c]	mf	mf	-	-	-	-
	ECR-Short – Adult attachment	mf	-	-	-	-	-
Parenting and work-family pressure	PPI – Parenting style	-	mf	-	-	mf	-
	PSS – Perception of parenthood	-	mf	mf	mf	mf	mf
	BPB – Experience of the parenting role	-	-	-	-	-	mf
	VIA – Migrants’ orientations toward mainstream and heritage traditions [c]	mf	mf	-	-	-	-
	Flexibility of working hours; QPSNordic – Work hours and work responsibilities; WAFCS – Work-Family pressure	-	mf	-	-	mf	mf
Covid-19-related questions	Conflict/support in the family due to the pandemic	-	-	mf	mf	mf	mf
	Changes in everyday family life, schooling situation, work-life, and social activities under to the pandemic, including use of digital tools and social media	-	-	mf	mf	mf	mf
	Changes in organisation of child-care [b] and child time-sharing agreement under to the pandemic [a]	-	-	mf	mf	mf	-
	Problems with housing, job security, economy, or alcohol/drug use	-	-	mf	mf	mf	mf
	Child emotional reaction to the pandemic	-	-	mf	mf	mf	mf
	Parent emotional reaction to the pandemic	-	-	mf	mf	-	-
	Pandemic burden in closest family and contact with welfare services	-	-	mf	mf	mf	-
Background (parent)	Year of birth and gender	mf	mf	mf	mf	mf	mf
	Birth country and year immigrated to Norway (if immigrated) [c]	mf	mf	-	-	-	-
	Adopted (from Norway/other country)	-	mf	-	-	mf	-
	Employment situation (TOPP, MoBa)	mf	mf	-	-	mf	mf
	Education level	-	mf	-	-	mf	mf
	Number, age, and gender of children with the other parent/with previous partner/with current partner	mf	mf	-	-	mf	mf
Background (child [a])	Age and gender	mf	mf	mf	mf	mf	mf
	Birth country (if not Norway) and age at immigration to Norway [c]	mf	mf	-	-	-	-
Background (family)	Residential status (parents cohabiting/separated/about to separate/never living together/parent widowed)	mf	mf	mf	mf	mf	mf
	Year of start of relationship with the other parent	mf	mf	-	-	-	-
	Married/cohabiting with the other parent (now/before break-up)	mf	mf	-	-	mf	mf
	Who initiated the parental break-up [a]	mf	-	-	-	-	-
	Duration since break-up [a]	mf	mf	-	-	mf	mf
	Distance between homes and who kept the common property [a]	-	mf	-	-	mf	mf
	Family financial situation (TOPP)	mf	-	mf	mf	mf	mf
	Use of family welfare services	mf	mf	-	-	mf	mf
	Experience with the family welfare service (from a survey used by the Family Welfare Services)	-	mf	-	-	mf	-
	Use of other help services and who required help	mf	mf	mf	mf	mf	mf
Re-partnering (self and other parent) [b]	Parent has new partner or not	mf	mf	-	-	mf	mf
	Year of relationship start with new partner	mf	mf	-	-	mf	mf

	Cohabit with new partner or not, new partner’s children (if any)	-	mf	-	-	mf	mf
	CPS – Conflict frequency, resolution; support from partner (TOPP); involvement and relationship between new partner and the parent’s child(ren)	-	mf	-	-	mf	mf
	Other parent has new partner or not; involvement of new partner in the parenting collaboration; half- and stepsiblings in the new family [c]	-	-	-	-	-	mf

Note. m = administered to mothers; f = administered to fathers. ASQ[1 2] = Ages and Stages Questionnaires; AUDIT[3] = The Alcohol Use Disorder Identification Test; BFI-44[4] = Big Five Inventory; CPRS[5 6] = Child-Parent Relationship Scale; CPS[7] = Conflicts and Problem-Solving Scales; EAS[8 9] = Emotionally, Activity and Shyness Temperament; ECR-Short[10] = The Experiences in Close Relationship Scale – Short; FAD[11] = Family Assessment Device; FSSQ[12] = The Duke – UNC Functional Social Support Questionnaire; GSAQ[13] = Global Sleep Assessment Questionnaire; PEQ[14] = Parental Environment Questionnaire; PPI[15] = Parenting Practice Inventory; PSS[16] = Parental Stress Scale; QPSNordic[17] = General Questionnaire for Psychological and Social Factors at Work; SCL[18] = Symptoms Checklist; SDQ[19] = Strengths and Difficulties Questionnaire; SIS[20] = The Security in the Interparental Subsystem Scales; SSIS[21] = Social Skills Improvement System; SWLS[22] = Satisfaction with Life Scale; VIA[23] = Vancouver Index of Acculturation; BPB[24] = Brief Parental Burnout Scale; WAFCS[25] = The Work-Family Conflict Scale; TOPP = Tracking Opportunities and Problems study, FORM = Divorce Mediation with High-Conflict Cases study; MoBa = the Norwegian Mother, Father and Child study.

[a] Only parents divorced/separated or about to separate.

[b] Asked separately for each child participating in the study (up to three of a maximum of five children).

[c] Only administered W1 or W2.

Supplemental Table 3

Overview of Study Domains and Instruments for Children 7 Years or Older

Domain	Instruments and description	W1	W2	W3 [a]	W4	W5	W6
Interparental conflict	CPIC – Perception of parental conflict	c	c	c	c	c	c
	SIS – Emotional security in the family context	c	c	-	-	c	c
Mediation and living situation [a]	Experience with mediation	c	-	-	-	-	-
	Importance of different aspects of living situation and parental cooperation	c	-	-	-	-	-
	Satisfaction with living situation	-	c	-	-	c	c
	Changes to living situation	-	-	-	-	-	c
Child mental health and wellbeing	KIDSCREEN – Health-related quality of life	c	c	c	c	c	c
	MFQ – Depressive symptoms	c	c	c	c	c	c
	SCARED – Anxiety symptoms	c	c	c	c	c	c
	Bullying (MoBa)	c	c	-	-	c	c
	School experience and screen time	-	-	-	-	-	c
	LoC – Locus of control	c	-	-	-	-	-
	SWLS – Satisfaction with life [b]	c	c	-	-	c	c
	SSRS – Social skills	-	c	-	-	c	c
	SDQ – Psychological symptoms of mental health [b]	c	c	c	c	c	c
	SCL – Psychological problems and difficulties [c]	-	-	-	-	-	c
	Experience with alcohol [b]	c	c	-	-	c	c
	Experience with other drugs [d]	-	-	-	-	-	c
	Sleep quality	c	c	-	-	c	c
Parent-child relationship	SS – Attachment security (rated separately for each parent)	c	c	-	-	c	-
	PEQ – Conflict in the parent-child-relationship (rated separately for each parent)	c	c	-	-	c	c
	Parent-child relation and communication	-	-	-	-	-	c
	FAD – General family functioning	-	c	c	c	c	c
	PQ – Parentification [b]	c	c	-	-	c	c
Other relationships	Relationship quality with siblings	-	c	-	-	c	c
	Relationship quality with parents' new partner	-	c	-	-	c	c
	Other persons in the child's support network	c	c	-	-	c	c
Covid-19-related questions	Conflict/support in the family and in the parent-child-relationship (rated separately for each parent)	-	-	c	c	c	c
	Changes in everyday life, school situation and social activities due to the pandemic, including use of digital tools and social media	-	-	c	c	c	-
	Changes in living situation due to the pandemic	-	-	c	c	c	-
	Emotional reaction to the pandemic	-	-	c	-	-	-
	Use of support network to cope with pandemic-related difficulties	-	-	c	-	c	-
	Infection/isolation/quarantine/hospitalisation within the closest family	-	-	c	-	c	-

Background (child)	Age and gender	c	c	c	c	c	c
	Birth country	c	c	-	-	c	-
Background (family)	Residential status (parents living together/apart; stepparents)	c	c	c	c	c	c
	Family constellations (full-, half-, and stepsiblings; parents' new partners)	-	c	-	-	c	c

Note. c = child report; CPIC[26] = Children's Perception of Interparental Conflict Scale; SIS[27] = Security in the Interparental Subsystem Scale; MFQ[28] = Mood and Feeling Questionnaire – Short form; SCARED[29] = Screen for Child Anxiety Related Emotional Disorders; LoC[30] = Locus of Control Questionnaire; SWLS[22] = The Satisfaction with Life Scale; SSRS[31] = Social Skills Rating System; SDQ[19] = Strengths and Difficulties Questionnaire; SCL[18] = Symptom Checklist; SS = Security Scale; PEQ[14] = Parental Environment Questionnaire; FAD[11] – Functional Assessment Device; PQ[32] = Parentification Questionnaire; MoBa = the Norwegian Mother, Father and Child study.

[a] Only children whose parents are divorced/separated or about to move apart.

[b] Only children ≥ 12 years.

[c] Only children ≥ 16 years.

[d] Only children ≥ 15 years.

Supplemental Table 4

Overview of Study Domains and Instruments for Children 0-6 Years (Childcare- or Schoolteacher Report)

**Domain**	**Instruments and description**	**W1**	**W2**	**W3**	**W4**	**W5**	**W6**
Child development and mental health	ASQ – Child development	t	t	-	-	t	-
	SISS – Social skills		t	-	-	t	-
	EAS – Child temperament	t	t	-	-	t	-
	SDQ – Emotional and behavioural problem screening	t	t	-	-	t	-
	Concerns about child	t	t	-	-	t	-
Childcare/ School	Child everyday life at childcare/school[30]	t	t	-	-	t	-
	Cooperation between childcare/school and home[30]	t	t	-	-	t	-
	Child reactions to separation and reunion with attachment figures (MoBa)	t	t	-	-	t	-
Covid-19-related questions	The pandemic's influence on child development	-	-	-	-	t	-
	The pandemic's influence on child everyday life in childcare/school	-	-	-	-	t	-
Background (child)	Age	t	t	-	-	t	-
Background (teacher)	Gender and duration of having known the child	t	t	-	-	t	-

Note. t = teacher report (childcare or school); ASQ[1 2] = Ages & Stages Questionnaires; EAS[8 9] = Emotionally Activity Shyness Temperament Questionnaire; SDQ[19] = Strength and Difficulties Questionnaire; SSIS[21] = Social Skills Improvement System; MoBa = the Norwegian Mother, Father and Child study.

References

1. Bricker D, Squires J, Mounts L, et al. Ages & Stages Questionnaire. Baltimore, MD: Paul H. Brookes, 1999.
2. Rasmussen L-MP, Martinussen M. Måleegenskaper ved den norske versjonen av Ages & Stages Questionnaire: Social and Emotional (ASQ: SE). *PsykTestBarn* 2013;2:1:1-6.
3. Babor TF, Higgins-Biddle JC, Saunders JB, et al. AUDIT: The alcohol use disorders identification test: Guidelines for use in primary health care, 2nd ed. Geneva: Switzerland: World Health Organization, 2001.
4. Engvik H, Clausen S-E. Norsk kortversjon av big five inventory (BFI-20). *Tidsskrift for norsk psykologforening* 2011;48(9):869-72.
5. Driscoll K, Pianta RC. Mothers' and fathers' perceptions of conflict and closeness in parent-child relationships during early childhood. *J Early Child Infant Psychol* 2011;7:1-24.
6. Pianta RC. Child-parent relationship scale. Unpublished measure, University of Virginia, 1992.
7. Kerig PK. Assessing the links between interparental conflict and child adjustment: The conflicts and problem-solving scales. *J Fam Psychol* 1996;10(4):454-73. doi: https://doi.org/10.1037/0893-3200.10.4.454
8. Mathiesen KS, Tambs K. The EAS Temperament Questionnaire—Factor structure, age trends, reliability, and stability in a Norwegian sample. *J Child Psychol Psychiatry* 1999;40(3):431-39.
9. Buss A, Plomin R. Temperament: Early Developing Personality Traits. Hillsdale, NJ: Lawrence Erlbaum 2008.
10. Brennan KA, Clark CL, Shaver PR. Self-report measurement of adult attachment: An integrative overview. In: Simpson JA, Rholes JA, eds. Attachment Theory and Close Relationships. New York, NY: Guildford Press 1998:46-76.
11. Epstein NB, Baldwin LM, Bishop DS. The McMaster family assessment device. *J Marital Fam Ther* 1983;9(2):171-80.
12. Broadhead W, Gehlbach SH, De Gruy FV, et al. The Duke-UNC Functional Social Support Questionnaire: Measurement of social support in family medicine patients. *Med Care* 1988:709-23.
13. Roth T, Zammit G, Kushida C, et al. A new questionnaire to detect sleep disorders. *Sleep Med* 2002;3(2):99-108. doi: https://doi.org/10.1016/S1389-9457(01)00131-9
14. Elkins IJ, McGue M, Iacono WG. Genetic and environmental influences on parent–son relationships: Evidence for increasing genetic influence during adolescence. *Dev Psychol* 1997;33(2):351-63. doi: https://doi.org/10.1037/0012-1649.33.2.351
15. Webster-Stratton C, Reid MJ, Hammond M. Preventing conduct problems, promoting social competence: A parent and teacher training partnership in Head Start. *J Clin Child Psychol* 2001;30(3):283-302. doi: https://doi.org/10.1207/S15374424JCCP3003_2
16. Berry JO, Jones WH. The parental stress scale: Initial psychometric evidence. *J Soc Pers Relat* 1995;12(3):463-72. doi: https://doi.org/10.1177/0265407595123009
17. Ørhede E, Hottinen V, Skogstad A, et al. User's guide for the QPSNordic: General Nordic Questionnaire for psychological and social factors at work. Copenhagen, Denmark: Nordic Council of Ministers 2000.
18. Derogatis LR, Lipman RS, Rickels K, et al. The Hopkins Symptom Checklist (HSCL): A self-report symptom inventory. *Behav Sci* 1974;19(1):1-15. doi: https://doi.org/10.1002/bs.3830190102
19. Goodman R. The Strengths and Difficulties Questionnaire: a research note. *J Child Psychol Psyc* 1997;38(5):581-86. doi: https://doi.org/10.1111/j.1469-7610.1997.tb01545.x
20. Davies PT, Forman EM. Children's patterns of preserving emotional security in the interparental subsystem. *Child Dev* 2002;73(6):1880-903. doi: https://doi.org/10.1111/1467-8624.t01-1-00512
21. Gresham F, Elliott S. Social Skills Improvement System (SSIS) Rating Scales Manual. Bloomington, MN: Pearson Assessments, 2008.

22. Diener E, Emmons R, Larsen R, et al. The life satisfaction scale. *J Pers Assess* 1985;49(1):71-75. doi: https://doi.org/10.1207/s15327752jpa4901_13
23. Ryder AG, Alden LE, Paulhus DL. Is acculturation unidimensional or bidimensional? A head-to-head comparison in the prediction of personality, self-identity, and adjustment. *J Pers Soc Psychol* 2000;79(1):49-65. doi: https://doi.org/10.1037/0022-3514.79.1.49
24. Aunola K, Sorkkila M, Tolvanen A, et al. Development and validation of the brief parental burnout scale (BPBS). *Psychol Assess* 2021;33(11):1125-37. doi: https://doi.org/10.1037/pas0001064
25. Haslam D, Filus A, Morawska A, et al. The Work–Family Conflict Scale (WAFCS): Development and initial validation of a self-report measure of work–family conflict for use with parents. *Child Psychiatry Hum Dev* 2015;46:346-57. doi: https://doi.org/10.1007/s10578-014-0476-0
26. Grych JH, Seid M, Fincham FD. Assessing marital conflict from the child's perspective: The Children's Perception of Interparental Conflict Scale. *Child Dev* 1992;63(3):558-72. doi: https://doi.org/10.1111/j.1467-8624.1992.tb01646.x
27. Davies PT, Forman EM, Rasi JA, et al. Assessing children’s emotional security in the interparental relationship: The security in the interparental subsystem scales. *Child Dev* 2002;73(2):544-62. doi: https://doi.org/10.1111/1467-8624.00423
28. Angold A, Costello EJ, Messer SC, et al. Development of a short questionnaire for use in epidemiological studies of depression in children and adolescents. *Int J Methods Psychiatr Res* 1995;5(4):237-49.
29. Birmaher B, Khetarpal S, Brent D, et al. The Screen for Child Anxiety Related Emotional Disorders (SCARED): scale construction and psychometric characteristics. *J Am Acad Child Adolesc Psychiatry* 1997;36(4):545-53. doi: https://doi.org/10.1097/00004583-199704000-00018 [published Online First: 1997/04/01]
30. Ruud T, Birkeland B, Faugli A, et al. Barn som pårørende. Resultater fra en multisenterstudie [Children with Ill Parents. Results From a Multi-Center Study]. Lørenskog: Akershus universitetssykehus HF, 2015.
31. Gresham FM, Elliot SN. Social Skills Rating System. Circle Pines, MN: American Guidance Service, 1990.
32. Jurkovic G, Thirkield A. Parentification Questionnaire. Atlanta, GA: Georgia State University, 1998.
