# Supplementary material for: Cohort profile: the Dynamics of Family Conflict (FamC) study in Norway
Source: BMJ Open. 2024 Aug 22;14(8):e080772. doi: 10.1136/bmjopen-2023-080772 (PMC11344520; doi:10.1136/bmjopen-2023-080772)
Supplement: Supplementary file 2 [file bmjopen-2023-080772.reviewer_comments.pdf]

PEER REVIEW HISTORY

BMJ Open publishes all reviews undertaken for accepted manuscripts. Reviewers are asked to complete a checklist review form (**http://bmjopen.bmj.com/site/about/resources/checklist.pdf**) and are provided with free text boxes to elaborate on their assessment. These free text comments are reproduced below.

ARTICLE DETAILS

**TITLE (PROVISIONAL)**	COHORT PROFILE: THE DYNAMICS OF FAMILY CONFLICT (FAMC) STUDY IN NORWAY
**AUTHORS**	Larsen, Linda; Buchmann, Nelli; Morbech, Maria; Holt, Tonje; Roysamb, Espen; Helland, Maren

VERSION 1 – REVIEW

**REVIEWER**	O'Hara, Karey<br>ASU REACH Institute
**REVIEW RETURNED**	07-Jan-2024

**GENERAL COMMENTS**	This study makes an important contribution to the literature. I appreciate the authors' thoughtful study design and their use of a longitudinal, multi-methods approach. This dataset will be used to address critical questions about family conflict and its impact on children. Although its findings may have limited applicability beyond the geographical area studied, it could inspire similar research elsewhere. My recommendations aim to improve the clarity and organization of the information.<br><br>1. The abstract states that 55% of parents were divorced/separated; does this imply the remaining 45% were married or cohabitating, albeit distressed as indicated by their need for counseling or therapy? Please confirm whether all participating families were distressed, as inferred from their attendance at the counseling center, regardless of divorce/separation status.<br>2. In the 'findings to date' section of the abstract, summarize the key findings rather than the research questions.<br>3. Maintain a consistent focus on families in reporting sample size. Present the number of families represented in each wave first, followed by the breakdown of family members participating. This dual-level approach will clarify the unit of measurement throughout the description. An alternative focus on individuals as the unit of measurement could be added to the supplemental materials.<br>4. Clarify the relevance of COVID-19 questions to the study's primary aims. If pertinent, retain the details within the cohort description. If not, consider deferring to a separate paper while providing a brief mention here.<br>5. In the variables section, specify what proportion of measures were validated and reliable versus those developed specifically for the study.<br>6. Consider merging the paragraphs discussing theory into one cohesive section that outlines the three theories foundational to the study.

	7. Consider reorganizing the introduction to focus first on the study's theoretical background, then the Norwegian context, and then the central research questions and the secondary research aims that have developed (including the COVID-19-related ones). Relocate the study's strengths to the appropriate section.<br>8. Add a "Location" subheading to the cohort description section, providing essential cultural information about Norway.<br>9. Simplify the explanation of the timelines for Waves 2 and 3 to enhance clarity.<br>10. Resolve the discrepancy between the 70% divorce/separation rate mentioned on page 10 and the 55% rate cited in the abstract. Clarify if 15% were contemplating separation.<br>11. Address the high attrition rates more thoroughly, including implications for the study and the nature of the missing data.<br>12. Clarify what is meant by 'strongest family attrition rate' – does it refer to the highest or lowest rate?<br>13. Include percentages in Table 2 for clearer understanding.<br>14. Rectify the inconsistency between the number of families recruited (2871) and those participating (2386) participated in one or more waves.<br>15. Consider restructuring the study variables section by covering an overview of the domains, administration methods, and the establishment of reliability and validity, with the details on pretesting and pilot testing included in the last part.<br>16. Strengthen the section discussing the Fam-C reference group with more details. I am not sure what the authors mean by "refer to the Cohort description."<br>17. Improve the 'findings to date' section with headings to group studies by their main focus and then a structured summary of each study, including sample specifics, research questions, and main findings.<br>18. Explain why the cohort is not representative of the general Norwegian population.<br>19. Please expand the collaboration section to detail the nature of the collaborations sought.<br>20. Consider combining Supplemental Tables 2, 3, and 4 to display measures with multiple reporters (e.g., ASQ: mft; SDQ: mfct) and denote in-house developed measures with a symbol for easy identification.

**REVIEWER**	Thompson, Morgan J.<br>University of Rochester
**REVIEW RETURNED**	12-Feb-2024

**GENERAL COMMENTS**	Thank you for the opportunity to review "Cohort Profile: The Dynamics of Family Conflict (Fam-C) Study in Norway." The authors describe an exciting study examining associations between interparental conflict and child development in the context of macro- and micro-level processes in a Norwegian sample. This study is an impressive longitudinal study with six waves of data, a large sample size, multiple reporters, and an extensive battery of assessments. The authors do an excellent job of detailing the study and highlighting key findings. I have a few clarifying points that I hope the authors will find useful as they move forward.<br><br>1. I appreciate the grounding in multiple theoretical frameworks (EST, family systems).<br><br>2. More information on Nordic welfare reforms and systems would be beneficial for understanding interparental conflict and even

	family conflict more broadly within the context of a Nordic welfare society. This addition is probably the result of me working at a US institution, but I think would help provide a better understanding of the study context and understand how effects of interparental conflict vary across societies.<br><br>3. In addition to stating the aims of Fam-C, more clearly stating the objectives or aims of the current manuscript in the Introduction would help guide the reader through.<br><br>4. The authors state that the main aim of Fam-C is to examine the interplay between macro and micro level processes to better understand relations between interparental conflict and child development. Some description of at least the primary proposed macro and micro level processes and linking these to theoretical frameworks in relation to interparental conflict and child development would provide very valuable information related to why these processes were assessed, their proposed role in research questions coming out of Fam-C.<br><br>5. As I was reading the information about the occurrence of each wave on page 8, I was trying to think of a way this could be presented more easily for readers then I looked at Figure 1 and this is fantastic! Very helpful figure!<br><br>6. The authors state “In some families neither the parents nor the children participated, but the teacher reported on one or more of the children (0-6 years) in the family.” This statement is a little confusing to me. It was possible for no family members themselves to report data? Did families still have to consent for teachers to provide data on the children?<br><br>7. I was looking for the Ns for the different variations of family members participating and found it in Table 2. It might be helpful to refer readers to this on page 8 or 9. There’s a lot to wrap your head around and seeing it in table format provided clarity.<br><br>8. I empathize with the challenges of working with parents/partners and tracking partnership changes. I commend the authors for tracking this and providing this information in such a clear manner.<br><br>9. I wonder if it would be helpful to examine whether attrition was related to any study variables or participant characteristics as well as if new participants at each wave differed from original study participants on any study variables or characteristics?<br><br>10. Figures 2 and 3 are very helpful!<br><br>11. I appreciate the transparency provided in supplemental tables 2-4 regarding the data available and who reported what at which waves.<br><br>12. I wonder if this work will be used for the authors to cite in papers coming out of Fam-C and if so, I wonder if it would be helpful to provide the internal consistencies of measures (supplemental tables 2-4)? I do not feel strongly about this and offer it more as a suggestion on the off chance the authors would see value in having that information readily available.

	13. Finally, the authors list attrition as a study weakness and although there was considerable attrition, the sample size is still relatively unmatched especially for studies focused on interparental conflict.

VERSION 1 – AUTHOR RESPONSE

Reviewer: 1
Dr. Karey O'Hara, ASU REACH Institute
Comments to the Author:
Thank you for doing this important work! This is an incredible dataset.
Review: BMJ Open-2023-080772 Title: COHORT PROFILE: THE DYNAMICS OF FAMILY CONFLICT (FAM-C) STUDY IN NORWAY This study makes an important contribution to the literature. I appreciate the authors' thoughtful study design and their use of a longitudinal, multi-methods approach. This dataset will be used to address critical questions about family conflict and its impact on children. Although its findings may have limited applicability beyond the geographical area studied, it could inspire similar research elsewhere. My recommendations aim to improve the clarity and organization of the information.

Response: We thank Dr O'Hara for taking time to read and comment on our paper, and also for her positive feedback and enthusiasm about our study. Dr O'Hare's thoughtful comments and suggestions have indeed helped strengthen our paper.

1. The abstract states that 55% of parents were divorced/separated; does this imply the remaining 45% were married or cohabitating, albeit distressed as indicated by their need for counseling or therapy? Please confirm whether all participating families were distressed, as inferred from their attendance at the counseling center, regardless of divorce/separation status.

Response: That is correct, the remaining 45% or so, were still living together. But just to clarify, we now state in the text that the "live together group" also includes families where parents were about to separate but had not yet done so.

Page 15: (includes also parents still living together but about to move apart).

We prefer not to label the families as distressed (or any term to that effect), but it would be fair to say that the families were help-seeking or experiencing challenges/going through a transition (i.e., divorce/separation). Words to that effect have been added to the Abstract.

2. In the 'findings to date' section of the abstract, summarize the key findings rather than the research questions.

Response: Thank you for this comment. We now showcase some of our findings in this section of the Abstract.

Page 2: Results show that interparental conflict patterns vary with family constellation. Interparental conflict severity is inversely related to the discrepancy between child- and parent-reported child-reactions to interparental conflicts, and child self-reported reactions is higher relative to parent-reported child-reactions. Other findings show that family characteristics (e.g., the number and age of children in the family, financial difficulties) are predictive of the type of residence arrangement that parents practice.

3. Maintain a consistent focus on families in reporting sample size. Present the number of families represented in each wave first, followed by the breakdown of family members participating. This dual-level approach will clarify the unit of measurement throughout the description. An alternative focus on individuals as the unit of measurement could be added to the supplemental materials.

Response: We are not quite sure, but think the reviewer refers to Table 2 (now Table 1) with this comment. We have now restructured this table so that the number of families participating at each wave is mentioned first, and then the individual participants mentioned after that.

4. Clarify the relevance of COVID-19 questions to the study's primary aims. If pertinent, retain the details within the cohort description. If not, consider deferring to a separate paper while providing a brief mention here.

Response: Thank you for alerting us that the FamC focus on Covid-19 needed better integration and explaining. We now briefly outline why the pandemic and its consequences for families is ideally studies within FamC and hence, why it became a new focus for us.

Page 8: Although the Covid-19 pandemic was not initially a focus of FamC, the cohort is ideally suited to addressing questions pertaining to the heightened concern for vulnerable families during and after the pandemic as it comprises help-seeking families and families going through divorce/separation, and includes pre-, peri- and post-pandemic measures. In the future, there are plans for even further follow-up data collections and cross-cultural comparison studies.

5. In the variables section, specify what proportion of measures were validated and reliable versus those developed specifically for the study.

Response: This is a valid comment, but not that straightforward to answer because it in part depends on which data collection wave one has in mind. As the Reviewer will probably appreciate, the surveys were quite complex in the sense that not all respondents (e.g., parents) received all the same questions. This is because some questions were only relevant for some participants (e.g., only parents living apart received question about residence arrangements and only parents that had re-partnered received question about their new partner). But to give a general estimate of how many measures (or items) were developed specifically for the study, we put this at 10%, but note that this figure is much higher for Wave 3 and Wave 4 (Covid-19 rounds). This is in part because there were no suitable/validated questionnaires available to assessing aspects related to the pandemic, but also because we wanted to assess things specific to the Norwegian context that necessitated that we develop our own measures/items.

Page 16: When validated questionnaires were not available, the research group developed appropriate items (estimated to be approximately 10% but varies slightly across data collection waves). For the Covid-19 waves a substantially higher proportion of researcher-developed items were used both to assess context specific pandemic effects but also because already validated questionnaires were not available.

6. Consider merging the paragraphs discussing theory into one cohesive section that outlines the three theories foundational to the study.

Response: Thank you for this comment. We have now merged the two sections that discuss the theoretical background for our study.

7. Consider reorganizing the introduction to focus first on the study's theoretical background, then the Norwegian context, and then the central research questions and the secondary research aims that have developed (including the COVID-19-related ones). Relocate the study's strengths to the appropriate section.

Response: We have followed the author guidelines for a BMJ Cohort Profile paper when structuring the manuscript including the Introduction and have therefore not restructured the Introduction as suggested by the Reviewer. We hope for the Reviewer's understanding.

8. Add a "Location" subheading to the cohort description section, providing essential cultural information about Norway.

Response: We have added a subheading as suggested. We have also added a little more information about the Norwegian context. We hope this covers what the Reviewer refers to as cultural information.

Page 8: Norway is geographically diverse and nearly half of the country's inhabitants live in the far south, around the capital Oslo or near the coastline in other large cities.

Page 8-9: Since its introduction in 1993, the quota for paid paternity leave has increased from four weeks to 10-14 weeks, with over 70% of fathers using their full paternity leave. Moreover, education (including university) is free of charge in Norway (with only few exceptions). Children start school the year they turn 6 and most attend secular public schools. Children generally attend the school closest to where they live, at least up to upper secondary school (ages 16-19), and while the school day is relatively short (about 8.30 am to 2pm), schools provide after-school care up to Grade 4 included. The social welfare system in the Nordic countries including Norway is generally more comprehensive and has more egalitarian cultural attitudes compared to for example, North America or the UK. These differing cultural contexts may lead to variations in how interparental conflict vary, is dealt with and impact family members across these societies.

9. Simplify the explanation of the timelines for Waves 2 and 3 to enhance clarity.

Response: We acknowledge that the timeline for Wave 2 and Wave 3 is complex and see that we unnecessarily explained this in too detailed a manner (although this was in fact how these two waves happened). We have thus rephrased it and now give a more succinct explanation.

Page 11: In April 2020, all families were invited to W3 irrespective of whether they had already been invited to W2. Steps were taken to keep the duration between W1 and W2 as similar as possible across participants, and thus, some participants received an invitation to W2 and W3 at the same time, while others received W3 before W2.

10. Resolve the discrepancy between the 70% divorce/separation rate mentioned on page 10 and the 55% rate cited in the abstract. Clarify if 15% were contemplating separation.

Response: The figures as stated in the text are correct. The 70% does not refer to families but rather children. That is, 70% of children who completed the survey at W1 had parents that are divorced/separated or about to separate. This is why the reported 70% differs to the 55% reported in the Abstract.

We have added the word "These" before describing the children, this makes it clearer that we are describing the children participating at W1 and not all the children enrolled in the study (i.e., the children with parental consent) or only those children whose parents participated at W1.

Page 12: Further, a total of 931 children from 692 families participated at W1. These children were on average 10.61 years (SD = 2.54) and slightly more girls (52.20%) than boys participated. More than 95% of children were born in Norway and almost 70% had parents that were divorced/separated or about to separate.

11. Address the high attrition rates more thoroughly, including implications for the study and the nature of the missing data.

Response: The Reviewer raises an important point and we have subsequently performed a series of sensitivity analyses to gauge if attrition is related to participant characteristics/key variables. As attrition was highest from Wave 1 to Wave 2, we focused on these two waves, and what we found was that attrition for mothers and fathers (respectively) was indeed related to their living situation (i.e., whether the parents live together or not). Specifically, among the parents who did not live together, the attrition rate was higher (i.e., they only participated at Wave 1). Moreover, for mothers only, attrition was also related to how many children she had with the father in the way that the attrition rate was higher among families with only one child. The parents' work situation, country of birth (Norway or other), life satisfaction (assessed with Satisfaction with Life Scale), and interparental conflict level was not predictive of attrition from Wave 1 to Wave 2.

Page 13: Attrition from W1 to W2 was related to the parents' living situation (i.e., live together or live apart) such that among the parents who did not live together, the attrition rate was higher (i.e., they only participated at Wave 1). Moreover, for mothers only, attrition was also related to how many children she had with the father in that the attrition rate was higher among families with only one child. The parents' work situation, country of birth, life satisfaction, and level of interparental conflict was not predictive of attrition from W1 to W2.

12. Clarify what is meant by 'strongest family attrition rate' – does it refer to the highest or lowest rate?

Response: We thank the Reviewer for picking this up. What we mean is of course highest. This has been corrected in the text. At the same time, we changed weakest [attrition] to lowest (same paragraph in the text).

13. Include percentages in Table 2 for clearer understanding.

Response: We totally agree with the Reviewer that it would enhance the understanding of the attrition rates (or on the flip side, participation rates) if percentages were included in Table 2 (now Table 1). However, to include these we would need to know how many participants were invited to participate at each data collection wave. Sadly, we do not have this information and nor can we access this retrospectively. The task of sending out the questionnaires (and generating the links to the questionnaires used during the interviews of children) was outsourced to a company external to our institute and information about how many participants were invited at each wave was not shared with us. We acknowledge that this is regrettable.

14. Rectify the inconsistency between the number of families recruited (2871) and those participating (2386) participated in one or more waves.

Response: We acknowledge that these numbers can be a little confusing, but they are in fact correct. A total of 2871 families were enrolled in the study when they attended a family counselling centre. This meant that (1) we could send them surveys to complete and (2) access register data on the families. But out of the 2871 families that were enrolled in the study (i.e., consented to the study), only 2386 completed one or more of the surveys they were sent. And we can still access the register data for families that have consented to the study even if they have not responded to any of the surveys. We hope this explanation clarifies why the number of recruited families differs from the number of families participating (i.e., completing the survey) at one or more waves.

In the text, we have added in brackets that by participating at W1, we mean completed the survey. Hopefully this helps with the interpretation.

15. Consider restructuring the study variables section by covering an overview of the domains, administration methods, and the establishment of reliability and validity, with the details on pretesting and pilot testing included in the last part.

Response: We thank the Reviewer for this comment. We have restructured this section so that we now have an overview of the study variables, then focus on the pretesting in children and then we describe our pilot project aimed at testing out the questionnaires on parents and children, and also psychometrically evaluating the key domains of interparental conflict and parent and child mental wellbeing using both parent- and child-report.

The restructured text is not included in the response letter, but we note that some minor changes to the wording in the text has been made.

16. Strengthen the section discussing the Fam-C reference group with more details. I am not sure what the authors mean by “refer to the Cohort description.”

Response: We thank the Reviewer for this comment. The section entitled Patient and public involvement has been strengthened and we have removed the last sentence referring to the Cohort Description as we did not feel this was particularly important. Just to clarify for the Reviewer, what we meant by Cohort description was simple the section entitled Cohort description.

Page 19-20: To enhance the feasibility and impact of FamC, a reference group was put together comprising high expertise researchers within the fields of family research and developmental psychology, and clinicians from the family counselling centres. The reference group was involved in discussions regarding the study design, the development of research questions and provided theoretical, methodological, and practical guidance on the project. The perspective of participants is deemed important to improve the quality, validity, and ethical conduct of FamC. Therefore, the participants can provide feedback on the questionnaires (or give other relevant information) using an open text field at the end of the survey. The implementation of study results in the practice field is undertaken in collaboration with Bufdir, and family counselling centres across Norway. Popular science summaries are provided on the project homepage for participants and the general population, including summaries specifically for the children.

17. Improve the 'findings to date' section with headings to group studies by their main focus and then a structured summary of each study, including sample specifics, research questions, and main findings.

Response: We thank the Reviewer for these helpful suggestions. However, according to the BMJ Cohort Paper author instructions this section should only provide a short summary of the most important results so far (rather than presenting the results) and refer instead to the relevant publications. We have therefore only made minor changes to the Findings to date section including adding subheadings as suggested by the Reviewer and adding a link to the project website where a list of all publications can be found.

18. Explain why the cohort is not representative of the general Norwegian population.

Response: We thank the Reviewer for prompting us to reiterate and clarify why the cohort is not representative of the general population. This has to do with the fact that families were recruited from family counselling centres across Norway when they attend mediation or sought help for other family/relationship matters. The text has been amended to reflect this information.

Page 23: Second, the FamC cohort is not representative of the general population in Norway, something that affects the generalizability of the study results. This is because the families were recruited when they sought help or attended mandatory mediation at a family counselling centre. The cohort can therefore be characterised as help-seeking and having some challenges in respect to family dynamics and relations, affecting its representativeness of the general Norwegian population.

19. Please expand the collaboration section to detail the nature of the collaborations sought.

Response: The Reviewer raises an important point here and we are thankful for the suggestion to expand the Collaboration section.

Page 23: The FamC cohort provides a plethora of research opportunities including, but not limited to: (1) longitudinal studies of interparental conflict and child development; (2) sibling studies focusing on siblings' differential experience and perception of interparental conflict, or the association between sibling relationship and interparental conflict; (3) studies focusing on fathers and father-child relationship; and (4) studies focusing on parental separation/divorce and different family constellations in relation to interparental conflict, and parent and child wellbeing. We particularly welcome collaborations that wish to utilize the longitudinal and multi-informant perspective as well as cross-cultural comparisons. We also welcome investigators to focus on the youngest children (below 7 years) in the cohort using the survey data collected from teachers and parents.

20. Consider combining Supplemental Tables 2, 3, and 4 to display measures with multiple reporters (e.g., ASQ: mft; SDQ: mfct) and denote in-house developed measures with a symbol for easy identification

Response: We thank the Reviewer for this suggestion, but after some discussion among the authors/study group, we have decided to keep Tables 2-4 unchanged due to the sheer complexity of the surveys. Moreover, some domains were assessed using a combination of validated measures and self-made items, which would make it difficult to denote with a symbol if a scale or measure was made in-house.

Reviewer: 2
Dr. Morgan J. Thompson, University of Rochester
Comments to the Author:
Thank you for the opportunity to review “Cohort Profile: The Dynamics of Family Conflict (Fam-C) Study in Norway.” The authors describe an exciting study examining associations between interparental conflict and child development in the context of macro- and micro-level processes in a Norwegian sample. This study is an impressive longitudinal study with six waves of data, a large sample size, multiple reporters, and an extensive battery of assessments. The authors do an excellent job of detailing the study and highlighting key findings. I have a few clarifying points that I hope the authors will find useful as they move forward.

Response: We would like to thank Dr Thompson for taking time to read and comment on our manuscript. She provides insightful comments and addressing these have helped lift and improve our manuscript. Thank you also for your enthusiasm and positivity regarding our study.

1. I appreciate the grounding in multiple theoretical frameworks (EST, family systems).

Response: Thank you.

2. More information on Nordic welfare reforms and systems would be beneficial for understanding interparental conflict and even family conflict more broadly within the context of a Nordic welfare society. This addition is probably the result of me working at a US institution, but I think would help provide a better understanding of the study context and understand how effects of interparental conflict vary across societies.

Response: A similar question was raised by the other reviewer (Comment 8). We have however lifted as suggested by the Reviewer here, that gender roles and quality, and the social welfare systems differ across the Nordic countries and for example the US, which in turn, may lead to variations in how conflicts are experienced and addressed within these differing cultural contexts. See changes to the manuscript text above under Comment 8.

3. In addition to stating the aims of Fam-C, more clearly stating the objectives or aims of the current manuscript in the Introduction would help guide the reader through.

Response: We thank the Reviewer for this comment and have now outline the aims of the cohort paper at the end of the first paragraph of the Introduction.

Page 4: In this cohort profile paper, we describe the background for the FamC study, give an overview of the cohort, and detail the study design to provide a basis for future analyses using the cohort. We also present a brief overview of some of the findings from the cohort to data.

4. The authors state that the main aim of Fam-C is to examine the interplay between macro and micro level processes to better understand relations between interparental conflict and child development. Some description of at least the primary proposed macro and micro level processes and linking these to theoretical frameworks in relation to interparental conflict and child development would provide very valuable information related to why these processes were assessed, their proposed role in research questions coming out of Fam-C.

Response: The Reviewer raises a good point. We now specify in the opening paragraph of the “Introduction” what we mean by macro and micro level processes. Further, when we return to the macro and micro level processes right before outlining the FamC project aims, we elaborate on the Norwegian cultural context in relation to North America (where most former research has been carried out), so as to provide a better foundation for the study aims. We hope this along with the new text added to the section now entitled “Location” sufficiently addresses the Reviewer’s concern/comment.

Page 5-6: Taking a cross-cultural perspective, family dynamics may be different in the Norwegian social democratic welfare context than in a more liberal welfare context such as North America. However, there is also evidence of some similarities. For example, in both Norway and the US there are negative associations between single motherhood and child adjustment with comparable moderation effects of family economic resources. Yet, little is known about whether findings regarding interparental conflict from North American studies generalize to Norway.

5. As I was reading the information about the occurrence of each wave on page 8, I was trying to think of a way this could be presented more easily for readers then I looked at Figure 1 and this is fantastic! Very helpful figure!

Response: We have simplified the information about each wave as suggested but the other reviewer. We acknowledge that it can be quite hard to follow how and when all the different waves occurred, especially since the pandemic forced us to re-jig things. But thank you for your enthusiasm about Figure 1, we are glad to hear it is helpful.

6. The authors state "In some families neither the parents nor the children participated, but the teacher reported on one or more of the children (0-6 years) in the family." This statement is a little confusing to me. It was possible for no family members themselves to report data? Did families still have to consent for teachers to provide data on the children?

Response: We thank the Reviewer for drawing out attention to this sentence. Some of the confusion may lie in the fact we talk of participation in the study but also participation in each data collection wave. Yes, it was possible for no family members to report data at one or more data collection waves, even if they were still consenting to be part of the study. And yes, the parents had to consent for the teacher to provide data on the youngest child(ren) in the family. This has been added to the Ethics section.

Page 15: Parents also consented for teachers to provide data on the youngest children (0-6 years).

The reason we included the sentence about neither parents nor children participating but teachers participating (and the couple of sentences preceding this) was only to show that any combination of family members and teacher could report data (i.e., participate) at a given data collection wave. But we see that this is confusing and have therefore removed this from the text. We have also moved the sentence about eligibility up to where recruitment is described. We hope these changes has improved the clarity of the text.

7. I was looking for the Ns for the different variations of family members participating and found it in Table 2. It might be helpful to refer readers to this on page 8 or 9. There's a lot to wrap your head around and seeing it in table format provided clarity.

Response: We appreciated that participation of families and participation by individual family members and teachers can be lot to wrap one's head around. We have moved Table 2 (now Table 1) up so that the Ns are presented in the section "Recruitment and data collection". We hope this provides better clarity.

8. I empathize with the challenges of working with parents/partners and tracking partnership changes. I commend the authors for tracking this and providing this information in such a clear manner.

Response: Thank you for this positive comment.

9. I wonder if it would be helpful to examine whether attrition was related to any study variables or participant characteristics as well as if new participants at each wave differed from original study participants on any study variables or characteristics?

Response: A somewhat similar question about attrition was raised by the other reviewer. See our response to Comment 11 above. We hope this adequately addresses the issue of study attrition.

10. Figures 2 and 3 are very helpful!

Response: Thank you.

11. I appreciate the transparency provided in supplemental tables 2-4 regarding the data available and who reported what at which waves.

Response: Thank you.

12. I wonder if this work will be used for the authors to cite in papers coming out of Fam-C and if so, I wonder if it would be helpful to provide the internal consistencies of measures (supplemental tables 2-4)? I do not feel strongly about this and offer it more as a suggestion on the off chance the authors would see value in having that information readily available.

Response: We understand why the Reviewer suggests including internal consistencies in Supplementary Tables 2-4, as we indeed do plan to cite our cohort profile paper in new papers coming out of FamC. However, we have decided not to do this as it would make the Tables even more complex, and we always provide internal consistencies in the papers that we write based on the FamC cohort. Further, we often use subsamples of the FamC cohort, like only focusing on families living apart, and if we were to provide internal consistencies for the various measures using the total FamC cohort and the different subsamples we use, that would definitely clutter the Supplementary Tables 2-4.

13. Finally, the authors list attrition as a study weakness and although there was considerable attrition, the sample size is still relatively unmatched especially for studies focused on interparental conflict.

Response: We are not quite sure if the Reviewer suggests here to remove attrition as a weakness of the study. We still include attrition as a weakness but now also state on page 20 that the sample size is relatively unmatched in studies focusing on interparental conflict as suggested by the Reviewer.

VERSION 2 – REVIEW

**REVIEWER**	O'Hara, Karey<br>ASU REACH Institute
**REVIEW RETURNED**	02-May-2024

**GENERAL COMMENTS**	I would like to commend the authors on their resubmission. The revisions have significantly enhanced the manuscript, making it a more substantial contribution to the field. The feedback from the previous reviews has been well addressed. I have a few remaining comments and suggestions for further refinement:<br><br>1. Typographical Errors and Clarifications:<br>- Page 4, Line 29: Minor typo - "data" should be corrected to "date".<br>- Page 6: Please confirm whether "inter-family" is the intended term. It seems like "inter-family" (between families) might be more appropriate than "intra-family" (within a family), based on the context provided.

	- Page 8: The text mentions that interviews for Waves 2, 3, 5, and 6 were conducted virtually, yet it is stated that no interviews occurred at Wave 5. This contradiction needs clarification.<br>2. Data Representation and Sample Size Descriptions:<br>- General: The description of the sample size, particularly in Table 1, remains unclear. This is my biggest lingering concern about the paper.<br>- Table 1: It may be beneficial to include teacher report samples under the 'Families' subheading within Table 1, rather than in a note. Adding the total number of families directly beneath the 'Families' subheading, similar to the 'Participants' subheading, would aid in clarity. I'm wondering why data from therapists and registries are not included in the table.<br>- Discrepancies in Reported Numbers:<br>- The manuscript states that 3,192 parents participated at Time 1 (T1), but the numbers in the table (1,792 mothers + 1,353 fathers = 3,145) do not align. Similarly, the numbers for children (931 reported vs. 919 in the table) and teachers (658 reported vs. 647 in the table) also show discrepancies.<br>- There is a mismatch in the number of mothers and fathers reported in Table 2 compared to Table 1 (Mothers: 1,823 vs. 1,947; Fathers: 1,369 vs. 1,477).<br>- The total number of families recruited is noted as 2,871, yet only 2,386 families participated in one or more waves. A clarification on whether there were families recruited that did not contribute to the dataset would be useful. The total adds up to 3,401 families when combining the initial sample and additional families at each wave.<br>3. Interpretation of Findings:<br>- The "Findings to Date" section is much improved and provides clear insights. However, the term "those in more complex family constellations" needs further explanation for better understanding.<br>- A summary statement regarding the findings of Morbech et al. (29) would be a valuable addition for readers.<br>- Suggestion**:** Adding percentage values to the consort diagram could significantly enhance its interpretability and ease of understanding for readers.<br><br>Overall, the manuscript is well-prepared, and these minor adjustments could further improve its clarity and impact. Thank you for the opportunity to review this interesting and important paper.

**REVIEWER**	Thompson, Morgan J.<br>University of Rochester
**REVIEW RETURNED**	16-Apr-2024

**GENERAL COMMENTS**	Thank you for the opportunity to review the revised manuscript entitled “Cohort Profile: The Dynamics of Family Conflict (Fam-C) Study in Norway.” The authors did an excellent job addressing concerns and I appreciate the thoroughness of their revisions. I have no further comments.

VERSION 2 – AUTHOR RESPONSE

Reviewer: 1
Dr. Karey O'Hara, ASU REACH Institute
Comments to the Author:

I would like to commend the authors on their resubmission. The revisions have significantly enhanced the manuscript, making it a more substantial contribution to the field. The feedback from the previous reviews has been well addressed.

Response: We thank Dr O'Hara for these positive comments and for taking time to review our manuscript and providing insightful and helpful comments.

I have a few remaining comments and suggestions for further refinement:

1. Typographical Errors and Clarifications:
   - Page 4, Line 29: Minor typo - "data" should be corrected to "date".

Response: Corrected

   - Page 6: Please confirm whether "inter-family" is the intended term. It seems like "inter-family" (between families) might be more appropriate than "intra-family" (within a family), based on the context provided.

Response: Thank you for this comment. You raise an important distinction these two terms. We have decided to remove the word "intra" to make the aim a bit more broad and now just say "family mediators and moderators".

   - Page 8: The text mentions that interviews for Waves 2, 3, 5, and 6 were conducted virtually, yet it is stated that no interviews occurred at Wave 5. This contradiction needs clarification.

Response: Thank you for picking up this error. No interviews were conducted at Wave 5. The text has been amended.

2. Data Representation and Sample Size Descriptions:
   - General: The description of the sample size, particularly in Table 1, remains unclear. This is my biggest lingering concern about the paper.

Response: We acknowledge that the sample size issue is tricky because (1) mothers, fathers, up to five children from the same family, and teachers are used as reporters; (2) some participants may have "skipped" certain data collection waves even if they did not drop out of the study; and 3) children can change "reporter category" from teacher report (younger children) to child self-report (children 7 year and older). This makes it difficult to compare the sample size across waves and specifically the third point about "reporter category" change, complicates the matter of attrition rate.

The sample size can be thought of in different ways, and we consider it important to look at the sample size at the family level as well as the participant level. Further and as shown in Table 1, at the family level, whether a family has participated can be determined in different ways. In line with your suggestions below, we have made some changes to Table 1 including the table note and hope this sufficiently improve the sample size description.

Moreover, we acknowledge that the difference between being recruitment into the study and participation by which we mean answered the questionnaire can be somewhat confusing. In some places in the text where we initially used the term participated/participation we now instead use the term answered the questionnaire.

   - Table 1: It may be beneficial to include teacher report samples under the 'Families' subheading within Table 1, rather than in a note. Adding the total number of families directly beneath the 'Families' subheading, similar to the 'Participants' subheading, would aid in clarity. I'm wondering why data from therapists and registries are not included in the table.

Response: Thank you for these comments. We have now added two rows under the Families subheading; the first outlines the total number of families that have participated based on any family member participating or teacher participation (the teacher reports on children aged 0-6 years), and the second outlines the total number of families where there is only teacher participation and no family members participating (previously stated in the table note that has now been removed).

Therapists only participated at W1. This has now been added to Table 1.

We are not quite sure what is meant by adding the registries to Table 1. From the registries we gathered (a vast amount of) information that applies to all families and individuals who were recruited into the study. This information was not limited to the those who filled in the questionnaire. While an overview of the register data is interesting, we however believe that this does not fit well or easily within Table 1 and thus, we have not included this here. We do however, state that register data is available for all recruited families (page 15).

- Discrepancies in Reported Numbers:
    - The manuscript states that 3,192 parents participated at Time 1 (T1), but the numbers in the table (1,792 mothers + 1,353 fathers = 3,145) do not align. Similarly, the numbers for children (931 reported vs. 919 in the table) and teachers (658 reported vs. 647 in the table) also show discrepancies.

Response: Thank you for picking up these inconsistencies. These were due to some individuals with incomplete data at Wave 1 being included in the descriptive statistics (the participation rates are based on participants with complete data only at each wave). We have re-run the descriptive statistics using samples with complete data only, and this resulted in only minor changes. In the process, we discovered that we had duplicated the numbers for two of the variables for mothers (Table 2) and this was corrected in the process. See Table 2 and the text on pages 10-11 for the updated descriptive statistics.

- There is a mismatch in the number of mothers and fathers reported in Table 2 compared to Table 1 (Mothers: 1,823 vs. 1,947; Fathers: 1,369 vs. 1,477).

Response: After addressing the previous comment the number of mothers and fathers (and children and teachers) now match between Table 1 and Table 2 (i.e., 1792 mothers and 1353 fathers at W1). The 1947 mothers refer to the total number of mothers that participated at one or more waves. Some mothers did not participate (did not complete the questionnaire) at W1, but they participated at a later stage. This is why the total for mothers (1947) is greater than the number of mothers that participated at W1 (1792). The same logic follows for fathers, children, and teachers. The mismatch in numbers is therefore not an error.

We see that it is confusing to add the n = 1947 for mothers, n = 1477 for fathers and so on in Table 2. Thus, we have removed these numbers, but we still mention the numbers in the table note below.

- The total number of families recruited is noted as 2,871, yet only 2,386 families participated in one or more waves. A clarification on whether there were families recruited that did not contribute to the dataset would be useful.

Response: The discrepancy between 2871 and 2386 is because not all families recruited into the study ended up actively participating. That is, they received the questionnaire/s (unless they had withdrawn consent) but did not complete the questionnaire/s. When we say that they participated in one or more waves, we mean that they provided data (i.e., answered the questionnaire/s). We now state this more clearly in the text.

Page 12: Of the 2871 families recruited, there were 2386 families that participated (i.e., contributed with data by answering the questionnaire) at one or more waves and around one third participated at only one wave (n = 795, 33.32%).

- The total adds up to 3,401 families when combining the initial sample and additional families at each wave.

Families in total add up to 3401 (which is more than the 2871 families that were recruited) because some families are counted more than once when you add up the “new included families”. When we say “new included families” at each timepoint, we refer to any families recruited to the study that did not participate at the previous round. They may not have participated up until this given timepoint, or they may have participated but not at the timepoint right before. For example, new included families at W4 (n = 255) refer to families that did not participate at W3 but may have participated at W1 and/or W2, or they may not have participated before at all (but have not withdrawn consent). We hope this explains why you arrive at 3401 families when you add up W1 families and all the “new included families”. We now explain this in a figure note for Figure 2, see page 23.

Page 23: Note. Families participating at W1 and all new included families at W2 to W6 add to 3401 families. This exceeds the number of families recruited to the study (n = 2871) because some families are counted more than once when adding up "new included families". New included families at each wave (e.g., W4) are those families that did not participate in the previous wave (i.e., W3). These families may not have participated up until this point, or they may have participated in earlier waves but not in the one immediately preceding the current wave. By family participation we mean that any family member answered the questionnaire.

3. Interpretation of Findings:
 - The "Findings to Date" section is much improved and provides clear insights. However, the term "those in more complex family constellations" needs further explanation for better understanding.

Response: Thank you for this comment. More text has been provided to clarify what is meant by "more complex family constellations".

Page 17: For parents living apart though, those in more complex family constellations (i.e., where one or both parents have re-partnered) have more destructive conflict behaviours and poorer conflict resolution compared to less complex family constellations (i.e., neither parent has re-partnered).

 - A summary statement regarding the findings of Morbech et al. (29) would be a valuable addition for readers.

Response: The main findings of Morbech et al. have been included in the text.

Page 17: Focusing specifically on families where parents live apart, Morbech et al.[29] explored parent characteristics in relation to what type of residence arrangement parents chose for their child, with results showing an increased likelihood of practising a 50/50 arrangement (i.e., equal time with both parents) when parents had more and older children, had fewer financial difficulties and had separated more recently. When parents had younger children, they were more likely to practice asymmetric shared residence (i.e., more time with one parent).

 - Suggestion**:** Adding percentage values to the consort diagram could significantly enhance its interpretability and ease of understanding for readers.

Response: Thank you for this comment; however, we have decided not to include percentages in the consort diagram as we feel the figure note that we have now added is more useful for enhancing the interpretability of the consort diagram. Moreover, we are a little uncertain how we should include the percentages as we do not have available the total number of families enrolled in the study at each wave. Stated differently, we know how many were initially recruited (n = 2871), but families withdrew/dropped out along the way, and we have no record of how many families this concerned at each wave. It thus makes it hard to say what proportion of enrolled/recruited families participated at each wave. We hope for your understanding.

Overall, the manuscript is well-prepared, and these minor adjustments could further improve its clarity and impact. Thank you for the opportunity to review this interesting and important paper.

Response: We are thankful for these kind words.

Reviewer: 2

Dr. Morgan J. Thompson, University of Rochester

Comments to the Author:

Thank you for the opportunity to review the revised manuscript entitled "Cohort Profile: The Dynamics of Family Conflict (Fam-C) Study in Norway." The authors did an excellent job addressing concerns and I appreciate the thoroughness of their revisions. I have no further comments.

Response: Thank you for taking the time to review our manuscript again, and for the positive feedback.
